# The effect of on-site and on-call nurse on exclusive breastfeeding in two different hospital settings: a prospective observational cohort study

**DOI:** 10.1186/s13052-024-01743-5

**Published:** 2024-09-17

**Authors:** Giuseppe Latorre, Domenico Martinelli, Manuela Capozza, Francesca Maria Grosso, Nicola Laforgia, Maria Elisabetta Baldassarre

**Affiliations:** 1 Neonatology and Neonatal Intensive Care Unit, Ecclesiastical General Hospital F. Miulli, Acquaviva Delle Fonti, Italy; 2 Section of Neonatology and Neonatal Intensive Care Unit, Interdisciplinary Department of Medicine (DIM), University of Bari “Aldo Moro”, Bari, Italy; 3 Department of Biomedical Sciences for Health, University of Milan, Milan, Italy

**Keywords:** Breastfeeding, On-site nurse, On-call nurse, Rooming in, Mother-newborn dyad

## Abstract

**Background:**

Exclusive breastfeeding during postpartum hospitalization is very important for ensuring the success of breastfeeding at home. The aim of the study is to determine if the on-site nurse in rooming in improves exclusive breastfeeding ratio.

**Methods:**

We conducted a prospective observational cohort study to evaluate exclusive breastfeeding during the first three months of life in two Neonatology Units in the South of Italy with different hospital settings: Ente Ecclesiastico Miulli of Acquaviva delle Fonti with on-site nurse h24 (on-site group) and Policlinico of Bari with nurse available on call h24 from Neonatology Unit (on-call group).

**Results:**

A total of 564 mother-baby dyads were admitted from 3 January to 31 March 2018 (299 in on-site group and 265 in on-call group). In the overall population, exclusive breastfeeding rate was 76.4% at 90-days, confirming the role of nurse and rooming in, independently of modality of setting. Considering the way of delivery, in infants from cesarean section there were higher rates for exclusive breastfeeding at 30 and 90 days of life in on-site group.

**Conclusions:**

We can assume that the presence of a nurse h24 could better identify breastfeeding problems. Our study suggests the role of on-site nurse during rooming in to encourage exclusive breastfeeding until three months of life in mothers who underwent caesarean section.

## Background

Breastfeeding has consistently been shown to be an exceptional preventive intervention for infants and mothers alike. The “personalized medicine provided by human milk” includes protection against morbidity and mortality, higher intelligence, and probable reduction in obesity and diabetes for breastfed children compared to children who are not breastfed [1]. In regard with obesity, breastmilk seems to have a protective effect, because of a reduced percentage of proteins and the presence of leptin, insulin, GLP-1 (Glucagon-like peptide-1), gastrointestinal peptide, and adiponectin, directly involved in the mechanisms of metabolic regulation and hunger/satiety balance [2].

Long duration of breastfeeding could reduce the risk of overweight, mainly in children not exposed to antibiotics during the breastfeeding period [3]. The long-term metabolic benefits of breastfeeding are conveyed by the intestinal microbiota, because different intestinal flora influences the metabolism of fatty acids and insulin sensitivity [4]. Breastfeeding could protect against obesity both in term and in preterm newborns, but it is important also for other at risk categories (e.g. low birth weight infants) who present a higher predisposition to endocrine or other chronic diseases, due to epigenetic mechanisms. [5] For nursing women, breastfeeding reduces the risk of breast cancer, improves birth spacing, and might also reduce the risk of ovarian cancer and diabetes. [6, 7]

There are few real contraindications to breastfeeding. Often some health conditions that can simply hinder the initiation and duration of breastfeeding are mistakenly considered to be real contraindications. Often the major determinants of a woman’s final choice of whether to nurse her infant or not are the attitude of health professionals and the mother's degree of information and awareness [8].

The American Academy of Pediatrics recommends exclusive breastfeeding for the first 6 months of life, with continued breastfeeding alongside the introduction of complementary foods for at least 1 year. [9] Global breastfeeding rates remain far below international targets, particularly in high-income countries. Data from the Center for Disease Control and Prevention's 2020 Breastfeeding Report Card indicate that less than 50% of infants in the United States were exclusively breastfed through 3 months. [10, 11] Data from WHO recommendations show only in Finland mothers do breastfeed their babies “exclusively” for six consecutive months [12].

There is often a gap between the mother's intention to breastfeed and feeding practices. Various aspects can influence the mother's desire to breastfeed, such as psychological, economic and social factors, as well as different nursing supports during the first days after delivery [13, 14]. Although the majority of nurses had a specific breastfeeding education, many women are seeking breastfeeding information online. Inadequate supply or difficulty feeding at the breast are often the reasons women cite for breastfeeding discontinuation in online-forum [15, 16]. Education and training of healthcare professionals in the knowledge and skills related to breastfeeding are very important and seem to be positively associated with improved nurses’ attitudes towards breastfeeding. [17] On the other hand, a positive relationship among mothers and nursing staff is correlated with early initiation of breastfeeding, even in preterm infants [18]. It is very important women receive this type of information as early as possible, even before birth. Many mothers feel that more support and guidance from hospital or community staff and family would have helped them to continue breastfeeding for longer. Mothers highlight specifically the lack of skilled advice and support for attachment and latching as a key factor in premature cessation of breastfeeding [19, 20]. Some studies showed that specialized health professionals could act as “breast feeding promoters” facilitating early initiation and reducing the abandonment at the first difficulties [21]. Since exclusive breastfeeding during postpartum hospitalization provides an important foundation for ensuring continued successful exclusive breastfeeding at home, support for breastfeeding in the first moments after childbirth increases the number of mothers who will then continue with exclusive breastfeeding [21, 22].

The range of different rates of initiation and continuation of breastfeeding in different settings demonstrates that the key factors influencing infant feeding rates are likely to be sociocultural and related to norms, public policy, and the availability of appropriate care and support [23, 24]. Awareness of which are the modifiable determinants affecting breastfeeding is essential in managing and supporting the breastfeeding dyad [25, 26] [27]

The aim of this study is to determine if the continuous presence of a nurse in the Rooming-in Unit, who provides information and answers mothers’ questions, could be a better practice than the presence of nurse available on call and improves exclusive breastfeeding ratio.

## Methods

We conducted a prospective observational cohort study to evaluate exclusive breastfeeding during the first three months of life in mother–child dyads in two Neonatology Units in the same geographical area, Apulia region, Southern Italy but with different hospital settings: Ente Ecclesiastico Ospedale Generale Miulli of Acquaviva delle Fonti, with on-site nurse h24 in the Nurse Unit (on site group) and Policlinico of Bari, with nurse not to be present in Nurse Unit but available on call h24 from Neonatology Unit which was on a different floor of the hospital (on call group).

Both Hospitals follow the steps 2, 5, 6, 7, 8, 9 of the Baby-Friendly Hospital Initiative. [28] The ten Steps to Successful Breastfeeding are as follows:

1. Have a written breastfeeding policy that is routinely communicated to all healthcare staff
2. Train all health-care staff in the skills necessary to implement this policy
3. Inform all pregnant women about the benefits and management of breastfeeding
4. Help mothers initiate breastfeeding within a half-hour of birth
5. Show mothers how to breastfeed and how to maintain lactation, even if they are separated from their infants
6. Give newborn infants no food or drink other than breast milk, unless medically indicated
7. Practise rooming-in – allow mothers and infants to remain together – 24 h a day
8. Encourage breastfeeding on demand
9. Give no artificial teats or pacifiers (also called dummies or soothers) to breastfeeding infants
10. Foster the establishment of breastfeeding support groups and refer mothers to them on discharge from the hospital or clinic.

In particular, step 2, which requires the training of all healthcare staff, represents an important intervention that improves breastfeeding rates. A recent systematic review, which included studies in 5 countries with 390 subjects belonging to the category of healthcare professionals, showed the usefulness of the action of healthcare personnel. [1] Provision of educational interventions aimed at increasing knowledge and practice of Baby-Friendly Hospital Initiative and support was found to improve health worker's knowledge, attitude, and compliance with the optimal breastfeeding practices. [1]

Both local Ethics Committees approved the study protocol.

Consecutive mother-baby dyads admitted to the units from 3 January to 31 of March 2018 were considered.

Healthy term newborns with gestational age ≥ 37 weeks in “rooming in” (baby in the same room of the mother all day) from birth to discharge and never hospitalized in Neonatal Intensive or Sub-Intensive Care Unit were included.

Exclusion criteria were all maternal and/or neonatal conditions that could interfere with breastfeeding (maternal HIV or active tuberculosis infection, herpes simplex lesions on both breasts, use of therapeutic radioactive isotopes, or exposure to radioactive materials, the use of drugs contraindicated in breastfeeding, galactosemia of the infant) or women not speaking Italian to ensure a full understanding of the questionnaire. Informed consent was obtained from both parents.

At discharge, a structured interview was performed, and a questionnaire was administered to the mother. The variables investigated with the questionnaire included sociodemographic features (maternal age and education), previous experiences (participation to a prenatal class and previous pregnancy), type of delivery, use of pacifier, nipple fissures, and satisfaction of nurse support. Variables subjected to changes during the timeframe of the study were collected by phone interview at 30 and 90 days of newborn’s life. The mode of breastfeeding was defined according to World Health Organization (WHO). The definition of breastfed infant was “an infant receives only breastmilk, no other liquids or solids are given”. [29]

During the study period, 400 infants were born in Ente Ecclesiastico Miulli Hospital: 43 preterm newborns with gestational age < 37 weeks and 51 newborns not exclusively assisted in rooming were excluded. A total of 303 dyads met the eligibility criteria. Among those, 7 were excluded (2 declined to participate and 5 did not speak Italian). In this hospital the Rooming-in Unit was continuously staffed by nurses that have additional training as lactation consultants, and who were physically present in the ward (on site group).

425 infants were born in Policlinico Hospital: 109 preterm newborns with gestational age < 37 weeks and 48 newborns not assisted in rooming-in were excluded. A total of 268 dyads met the eligibility criteria. Among those, 3 were excluded because did not speak Italian. In Policlinico Hospital a nurse with additional training as lactation consultant was available on call when a mother needed help for breastfeeding or to answer to her questions but was not physically present in the mother’s hospital ward (Rooming in Unit); they guaranteed a round in the Rooming in Unit two times a day. For the day long, a relative was present in the same room of the mother-infant dyads to take care of mother and newborn (on call group).

So, a total of 564 mother-baby dyads were examined, 299 in the on-site group and 265 in the on-call group.

In both groups no mothers reported post-partum depression, flare-up of previous breast pathologies (excluding fissures) or problems related to the family or external environment. 13/299 mothers (4,3%) in the on-site group and 35/265 (13,2%) in the on-call group reported little and/or discordant information about breastfeeding received during hospitalization. Of these, 6/13 (46,1%) in the on-site group and 17/35 (48,5%) in the on-call group were not exclusively breastfeeding at 1 and 3 months.

Data were collected by a healthcare professional at discharge and/or extracted from infants’ computerized medical charts (Neocare, I&T Informatica e Tecnologia Srl, Italy) and by phone interview at 30 and 90 days of newborn’s life. One mother did not answer at 30 days interview and 46 at 90 days and were excluded in the analysis of 30 and 90 days respectively.

Data were reported as mean ± standard deviation or percentage for categorical variables. The Student's t-test was used to compare continuous variables. Associations between categorical data were evaluated by using Chi-squared test or Fisher Exact test as appropriate. Multivariate logistic regression model was used to adjust for possible confounder factors. For all data a p-value of 0.05 or less was considered statistically significant. All analyses were conducted using STATA software, version 16 (Stata-Corp LP, College Station, Texas, USA).

## Results

Questionnaires showed differences between cohorts (on site vs. on call group) in terms of maternal education, prenatal class attendance, pacifier usage, weight at birth and at discharge. In on call group of Policlinico Hospital there was a greater proportion of mothers with primary school and less frequent university education, a lower percentage of prenatal class, more recurrent pacifier usage and a slight greater weight compared to on-site group of Ente Ecclesiastico Miulli Hospital. Characteristics of mothers and infants in the two study groups are reported in Table 1.

**Table 1:** Characteristics of mothers and infants in the two study groups

	**On-site**	**On-call**	
	*n* = 299	*n* = 265	p
Maternal age at delivery (years)	33 ± 5	33 ± 5	0,407
**Primary school**	**7,8%**	**19,6%**	**0,000**
Secondary education	56,4%	56,2%	0,963
**University degree**	**35,8%**	**24,2%**	**0,003**
Parity 1	51,2%	48,3%	0,496
Parity 2	48,8%	51,7%	0,496
**Prenatal class**	**50,8%**	**40,4%**	**0,013**
Nipple fissure	31,2%	37,0%	0,149
**Pacifier use**	**43,0%**	**58,5%**	**0,000**
**Mother satisfaction**	**95,6%**	**84,5%**	**0,008**
Female infant	53,8%	49,1%	0,256
Male infant	46,2%	50,9%	0,256
Gestational age (weeks)	39.3 ± 1.1	39.2 ± 1.2	0,196
Vaginal delivery	72,9%	72,1%	0,825
Caesarean section	27,1%	27,9%	0,825
**Weight at birth (g)**	**3344 ± 417**	**3462 ± 394**	**0,001**
**Weight at discharge (g)**	**3141 ± 396**	**3250 ± 386**	**0,001**
Weight loss (%)	6.0 ± 2.9	6.1 ± 2.5	0,665

**Fig. 1:**
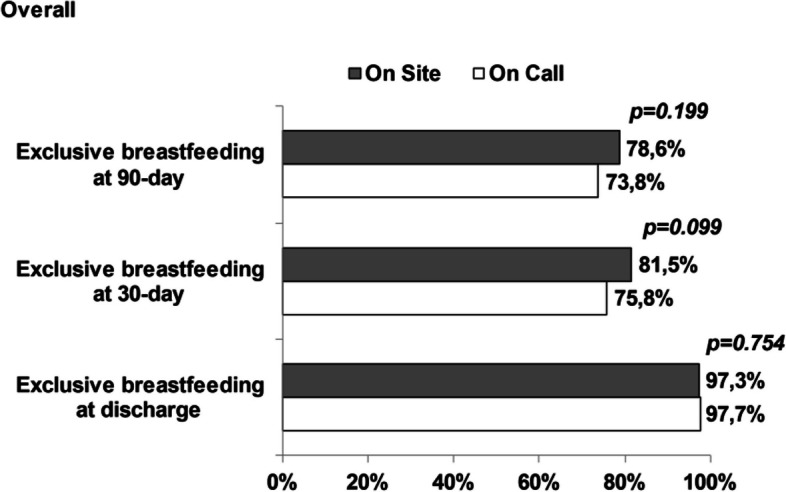
Breastfeeding proportion in overall population, in on-site and on-call groups

In the overall population, exclusive breastfeeding was observed in 550 dyads at discharge (97.5%), 444 dyads (78.9%) at 30 days and 396 dyads (76.4%) at 90-days. The shift on the non-exclusive breastfeeding was detected in 16 (2.7%) at discharge, 117 (19.9%) at 30-day and 48 (8.2%) at 90-day. No differences regarding breastfeeding proportion were detected between on site and on call groups at discharge (97.3% and 97.7%, respectively). There was a significant better proportion of exclusively breastfed infants in on site group respect to on call group at 30 days (81.5% vs. 75.8%, respectively) and 90 days (78.6% vs. 73.8%, respectively). Breastfeeding proportion in on site and on call group are reported in Fig. 1.

We divided the overall population in two groups by way of delivery: vaginal delivery group and cesarean section group. Table 2 shows characteristics of mothers and infants in the two study groups stratified for way of delivery.

**Table 2:** Characteristics of mothers and infants in the two study groups stratified for mode of delivery

		**Vaginal**			**Cesarean**	
	On-site	On-call		On-site	On-call	
	*n* = 218	*n* = 191	p	*n* = 81	*n* = 74	P
Maternal age at delivery (years)	32 ± 5	33 ± 5	0,322	34 ± 5	34 ± 6	0,968
**Primary school**	**6,5%**	**18,3%**	**0,000**	**11,1%**	**23,0%**	**0,048**
Secondary education	59,1%	56,0%	0,535	49,4%	56,8%	0,358
University degree	34,4%	25,7%	0,055	**39,5%**	**20,3%**	**0,009**
Parity 1	55,0%	50,3%	0,334	40,7%	43,2%	0,752
Parity 2	45,0%	49,7%	0,334	59,3%	56,8%	0,752
**Prenatal class**	**52,8%**	**41,4%**	**0,021**	45,7%	37,8%	0,323
Nipple fissure	30,9%	35,1%	0,367	32,1%	41,9%	0,207
**Pacifier use**	**43,3%**	**60,7%**	**0,000**	42,0%	52,7%	0,181
**Mother satisfaction**	**95,4%**	**85,9%**	**0,001**	**96,3%**	**81,1%**	**0,002**
Female infant	55,0%	48,7%	0,199	50,6%	50,0%	0,939
Male infant	45,0%	51,3%	0,199	49,4%	50,0%	0,939
Gestational age (weeks)	39.5 ± 1.0	39.3 ± 1.2	0,044	38.7 ± 1.2	38.9 ± 1.3	0,489
**Weight at birth (g)**	**3328 ± 41**	**3476 ± 38**	**0,000**	3388 ± 42	3423 ± 41	0,607
**Weight at discharge (g)**	**3134 ± 40**	**3265 ± 37**	**0,001**	3162 ± 38	3210 ± 42	0,459
Weight loss (%)	5.8 ± 2.9	6.1 ± 2.3	0,322	6.6 ± 2.8	6.3 ± 2.9	0,463

In vaginal delivery group there were differences between cohorts in terms of maternal education, prenatal class attendance, pacifier usage, weight at birth and at discharge, and gestational age. In on call group, compared to on-site group, there was a greater proportion of mothers with primary school and less frequent university education, a lower percentage of prenatal class, more recurrent pacifier usage and a slight greater weight.

In Cesarean section group there were differences between cohorts in terms of maternal education, prenatal class attendance, satisfaction of nurse assistance during recovery. In on call group, compared to on-site group, there was a greater proportion of mothers with primary school and less frequent university education, a lower percentage of satisfaction about nurse assistance.

**Fig. 2:**
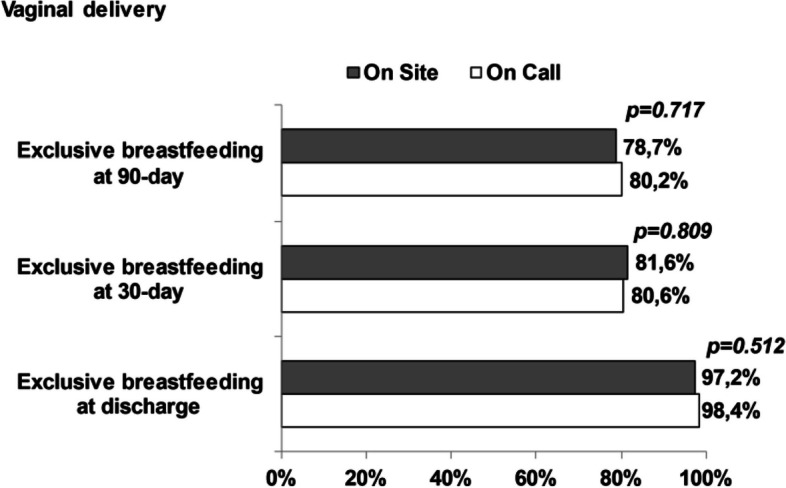
Breastfeeding proportion in vaginal delivered infants in on-site and on-call groups

**Fig. 3:**
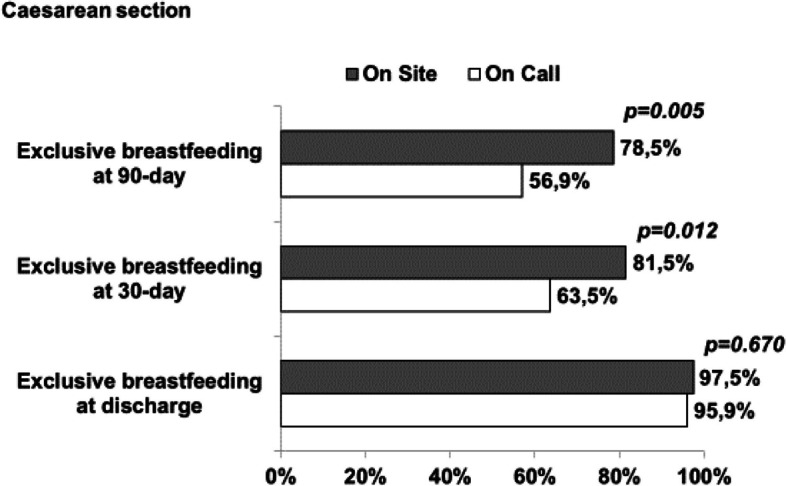
Breastfeeding proportion in caesarean section delivered infants in on-site and on-call groups

No statistical differences were found between on site and on call group in infants born from vaginal delivery. In newborns from cesarean section, in on site group there were higher rates for exclusive breastfeeding at 30 and 90 days of life than in on call group, while at discharge there was a similar proportion (Figs. 2 and 3). Statistical differences in Cesarean section group, both at 30 days and 90 days of infants’ life, still remained when adjusted for education (*p* < 0.05) and mother satisfaction of nurse (*p* < 0.05).

## Discussion

Breastfeeding provides short-term and long-term health, economic, and environmental advantages to children, women, and society. Supporting the breastfeeding dyad, has not only health benefits, but also social implications. [30] Various support measures, at many levels, from legal and policy directives to social attitudes and values, to women's work and employment conditions, to health services, could rapidly improve breastfeeding rates [31].

There are many studies to support the fact that rooming-in increases breastfeeding rates. The practice of rooming-in as defined by the World Health Organization and United Nations Children’s Fund is a “hospital practice where postnatal mothers and normal infants stay together in the same room for 24 h a day from the time they arrive in their room after delivery”. [20] In this study rooming in is implemented in both centers with the only difference of way of nurse support. Pediatric nurse practitioners rated as slightly important the limited time during health supervision visits to address breastfeeding problems and to give routine advice on breastfeeding. [21] The continuous presence of a nurse expert in breastfeeding support independently of on site or on call setting should reduce such limitations our study suggests. The nurse's role in support of breastfeeding varies with the time and place where patient care is provided. In each setting, however, the nurse plays a significant role in helping the mother to begin breastfeeding and to enjoy it, at the same time providing her infant with optimum nutrition for his early growth and development. [17] Postnatal breastfeeding counselling and support has been shown to increase rates of breastfeeding up to 6 months of age. [22]

To encourage breastfeeding, it is important to take care of mother before delivery, with dedicated preparation courses, and after delivery, with health care providers with specific competence, including trained midwives and neonatal nurses. A recent two years retrospective study showed a significant decrease of maternal and neonatal mortality/morbidity rates could be achieved by providing effective perinatal and newborn care also in high-income countries. [32]

The high rate of breastfeeding at three months we found in the studied population confirms the role of nurse and rooming in independently of modality of their setting in the rooming in spaces. Early advices to position and attachment of newborn to the nipple can prevent breastfeeding problems later. The stay in the facility providing maternity and newborn services is an opportunity to discuss and assist the mother with questions or problems related to breastfeeding and to build confidence in mother ability to breastfed independently of where the nurse is located.

Cesarean section is a well-known determinant of reduced exclusive breastfeeding rate. [23] The presence of an on-site nurse seems to increase the exclusive breastfeeding, after the discharge, at 30 and 90 days of life. We can assume that the presence of a nurse 24 h in the same ward of the mother could better identify breastfeeding problems in mother-newborn dyad, especially in case of cesarean section that could make the interaction between mother and child more difficult.

Many interventions have been recommended to promote and increase breastfeeding, including a previous successful breastfeeding experience, a higher level of education of the mother, attending prenatal classes, no use of pacifier, rooming in practice, and breastfeeding on demand. On the other hand, factors acting negatively on breastfeeding were advanced maternal age, non-spontaneous delivery, perception of low milk supply, mastitis, and nipple fissures. [24, 25] It is important to individualize the assistance provided to breastfeeding mothers, paying special attention to personal experiences.

## Conclusion

Our study suggests the role of on-site nurse during rooming in stay of the mother-infant dyad to encourage exclusive breastfeeding until three months of life in mother who underwent caesarean delivery.

A 24-h nurse, always present in the ward, ready to help the mothers, could be decisive for the "chain of solidarity" necessary to increase exclusive breastfeeding rates. However, this organization is not always possible. Organizational and management difficulties, which include the possible unavailability of a nurse on site 24 h a day, are often encountered in public hospitals. Many times the same nurses take care of rooming in and also sub-intensive or even neonatal intensive care, thus making communication with mothers and breastfeeding support during hospitalization more difficult.

More studies are needed to better understand our results.

## Data Availability

“Data will be made available on reasonable request”.

## Abbreviations

WHO: World Health Organization
IUGR: Intrauterin Growth Restriction
